# Novel Preparation and Characterization of Resol Resin with Phenolated Kraft Lignin

**DOI:** 10.3390/polym18141691

**Published:** 2026-07-09

**Authors:** Nina Žibret, Tine Vojska, Peter Bukovec

**Affiliations:** 1 Fenolit d.d., Synthetic Resins and Moulding Compounds, Breg 22a, 1353 Borovnica, Slovenia; tine.vojska@fenolit.si; 2 Faculty of Chemistry and Chemical Technology, University of Ljubljana, 1000 Ljubljana, Slovenia; peter.bukovec@fkkt.uni-lj.si

**Keywords:** lignin, phenolation, lignin–phenol–formaldehyde resin, emissions, tetradimer, flexural strength, thermal insulation

## Abstract

The application of lignin as a sustainable replacement for phenol in resin is one of the main priorities in the polymer industry. The partial substitution of phenol by Kraft lignin in the synthesis of resole resin, a mineral and glass wool insulation binder, was thus investigated. Lignin was activated by phenolation in an alkaline medium at low temperature, followed by reaction with formaldehyde in the same batch. Conducting the phenolation reaction in an alkaline medium allows the synthesis of resol resin to continue without interrupting the process, while the low temperature ensures the low viscosity of the synthesized resol, which is a prerequisite for its use as a binder in the manufacture of thermal insulation products. This is an important innovation that streamlines the production of modified resol. Activated lignin and resole resins were characterized by Fourier transform infrared spectroscopy (FTIR) and gel permeation chromatography (GPC). Phenolation occurs mainly via the binding of phenol to the lignin macromolecule, increasing the molecular weight of activated lignin, with only small amounts of low molecular weight species observed. Resol resins with and without incorporated lignin have identical FTIR spectra and similar molecular weight distributions, which confirms the successful synthesis of lignin-containing resin. With essential relevance for the undisturbed production of thermal insulation products, the most suitable of the resins synthesized with lignin has appropriate viscosity, double the stability of the reference product, and half the amount of tetradimer (tetradimer can cause problems due to precipitation). In addition, this resin results in significantly lower emissions and has increased flexural strength. The synthesis is transferable to industrial practice.

## 1. Introduction

Phenolic resins have been synthesized for more than a century [1], with a very broad range of applications, such as mineral wool insulation, binders, laminates, composites, and molding compounds [2]. There are two basic types of phenolic resins: resol and novolac. Resol is prepared under alkaline conditions, while novolac is prepared under acidic conditions [3,4,5]. Waterborne resoles of low molecular weight and appropriate concentration (5–20%) [2] exhibit good penetration properties in thermal insulation panel production [6], with the final products demonstrating long-standing thermal and mechanical stability [2]. The characteristics of the produced resoles, such as viscosity, pH, and B-time, depend on the final product’s specified use defined by the manufacturer or customer [1].

Phenol, the main component of resole synthesis, is produced from crude oil. The instability of the oil market and environmental consequences are forcing the polymer industry to search for cost-effective, environmentally friendly replacements for phenol [1].

Lignin, as a natural polyphenol, offers strong potential as a partial replacement for phenol in resins. It is the most abundant natural aromatic biopolymer on earth, obtained in large quantities as a side product in the paper and pulp industry. Kraft lignin, isolated in the alkali process for paper pulp production, is the most common and easily accessible form of lignin, and has often been used in laboratory experiments to replace phenol [6,7]. However, the complex and very stable structure of lignin results in poor chemical reactivity, representing the main obstacle to its industrial use in unmodified form [6,8,9]. The three phenylpropanoid monomers, coniferyl, sinapyl, and p-coumaryl alcohols, which constitute the macromolecule of lignin, are linked by carbon–carbon and ether bonds [6,7]. These linkages considerably reduce the number of reactive aromatic sites for formaldehyde addition [10,11,12].

A brief review by Hu et al. [10] outlines several methods that aim to enhance lignin’s reactivity, e.g., the demethylation of reactive aromatic hydroxyl groups, the incorporation of hydroxymethyl groups onto aromatic rings, the treatment of lignin with phenol, the reduction of the aldehyde and ketone units in lignin, lignin oxidation, and hydrolysis of lignin. The reaction of lignin with phenol called phenolation is one of the most widely used activation methods and can be carried out in an acidic or basic medium.

Data on the phenolation of lignin under alkaline conditions are quite scarce. Base-catalyzed hydrolysis of Kraft lignin was carried out by Solt et al. [13] at 250 bar and 320 °C, generating monomeric and oligomeric phenolic compounds. Resins prepared in this way have viscosities ranging from 220 to over 5000 mPa·s and are suitable adhesives for hot-pressing applications. Gan and Pan [9] phenolated Kraft lignin in an alkaline medium, at 160 °C in a laboratory glass reactor. They identified monomeric and dimeric phenolics and organic acids, which, in their opinion, have potential applications as platform chemicals. In a recently published study [14], the authors propose integrated lignin phenolation-kraft pulping process at 160 °C for co-producing phenolated lignin and kraft pulp in one pot, as a promising method of preparation of high-value chemicals and materials.

Our aim was to prepare a lignin-incorporated resole resin suitable as a binder for thermal insulation applications. Specific requirements for this type of resole resin include low viscosity (not exceeding 50 mPa·s), high water solubility, expressed as water tolerance (WT) and sufficient stability of the aqueous resole solution [1]. WT testing measures how much water a liquid resin can mix with before it turns cloudy or separates into layers. A WT of 1:25 should preferably be achieved in resole for mineral wool manufacturing and is closely related to resin stability. Storage stability relates to haze appearance upon storage over time, which is a consequence of polycondensation. It is accepted that a good-quality resin lasts at least two weeks. It is also mentioned in reference [1] that commercial resol resins usually contain 45 to 55% dry matter, which is optimal regarding transportation costs.

This work also examined the scale-up of resol resin synthesis to an industrial level. Hence, the sustainability of the process was considered from the very beginning of planning the stages of a stepwise synthesis. As previously mentioned, most phenolation experiments for Kraft lignin, cited in the literature, were carried out in acidic media and on a laboratory scale. The activation of Kraft lignin under these conditions and subsequent increase in pH to high values, as necessary for resol synthesis, would represent an unacceptable waste of reactants and time, increasing the cost of the final product. Furthermore, any separation, purification, and drying of the activated Kraft lignin prior to its dissolution in sodium hydroxide would also increase costs. We therefore decided to activate Kraft lignin in an alkaline medium and then continue with the synthesis of resole resin in the same reaction system, first in a laboratory vessel and then in a production-scale reactor. The focus was also on lower temperature processes for both steps, preferably at temperatures between 50 °C and 75 °C.

A specific problem in handling the resol binder used in insulating mineral wool is posed by [bis(4-hydroxy-3,5-dimethylolphenol) methane], usually referred to as tetradimer [1]. The issue resides in its possible partial precipitation from the aqueous resin solution, leading to problems in storage tanks, piping, and other equipment. Also, upon dilution of the clear resole solutions with water prior to their usage as a binder, precipitation of tetradimer may occur for reasons that are currently unknown. The reported concentrations of tetradimer in typical phenol–formaldehyde resins are usually quite high, e.g., 14–17% [1] and 10–18% [15]. In these studies, the authors used sulfides to alter the chemistry of methylolation and condensation, thereby reducing tetradimer formation. Additionally, reference [1] described other possibilities for overcoming this problem. To the best of our knowledge, the problem of tetradimer precipitation in the syntheses of lignin-modified resoles has not yet been reported. Due to its crucial role in the production of resole binders used in insulating mineral wool panels, we examined the concentrations of tetradimer in prepared resol solutions and monitored its possible precipitation.

In our study, we report a new method of resol resin synthesis with the partial replacement of phenol with lignin, where the base-catalyzed activation of lignin and resol synthesis are carried out in the same batch, without an intermediate interruption of the process. The effects of phenolation and the resin synthesis conditions on the final resole’s characteristics were examined and compared to those of conventional resoles. Monitoring the tetradimer concentration in lignin-modified resole shows a significant reduction compared to the unmodified resole, with a significant improvement in resin stability. The alkaline lignin phenolation processes described so far use high temperatures and pressures to depolymerize lignin into smaller fragments or even monomers. One of the major advantages of the present method is the low temperature used for activation of lignin with the addition of phenol without lignin depolymerization. The new synthesis route significantly improves the carbon footprint of lignin-modified resin.

## 2. Materials and Methods

### 2.1. Materials

Industrial Kraft lignin was used in this study. The product comprises 65–68% Kraft lignin, with a molecular weight of 4500–6000 g/mol. The moisture content of lignin was determined by oven drying at 105 °C to a constant weight. The concentration of raw materials used was as follows: 100% phenol, >99.5% NaOH, >99.5% ethanol, and 47–50% formaldehyde (which contains less than 100 ppm of formic acid and 0.55% methanol). All chemicals used were commercially available and of technical grade.

### 2.2. Water Miscibility

Water miscibility (WM), also called water tolerance (WT), was determined according to ISO standard method 8989 [16]. A total of 10 g of resin was added to 250 mL of distilled water at 23 °C, and the mixture was visually checked to determine whether it was turbid or clear. The resin has WT of 1:25 if the solution is clear after the resin’s addition and no change in opacity is visually detected.

### 2.3. Dry Content

The dry content was determined according to ISO standard method 8618 [17]. The dry content was measured to check the volatile matter in the resin by heating 3 g of resin at 135 °C for one hour. Under these conditions, the volatile substances were partially removed from the resin. From the difference in weights, the dry matter content of the non-volatile residue was calculated.

### 2.4. Free Phenol with Gas Chromatography (GC)

The free phenol content was determined by gas chromatography (GC) using ortho-cresol as an internal standard. A weighed amount (approximately 1 g) of sample and a recorded drop of ortho-cresol (≈0.02 g) were dissolved in methanol, the pH was adjusted to 7 using NaOH/HCl, and the solution was vortexed. One to two microliters of the final solution was injected into a GC–FID equipped with a DB-5ms column (30 m × 0.25 mm, 0.25 µm). The oven was programmed from 60 °C (1 min) to 250 °C at 10 °C min^−1^, with the injector and detector at 250–280 °C. Quantification was performed by internal standard calibration using phenol standards prepared under identical conditions.

### 2.5. Free Formaldehyde

The free formaldehyde in water-soluble resins was determined according to ISO 11402 [18] using the hydroxylamine hydrochloride method. The basis for this method is the titration of the hydrochloric acid that is liberated when hydroxylamine hydrochloride reacts with formaldehyde to form formaldoxime:

HCHO + NH_2_OH:HCl → CH_2_:NOH + H_2_O + HCl

The free formaldehyde in phenolic resins is present as monomeric formaldehyde, hemiformals, polyoxymethylene hemiformals, and polyoxymethylene glycols. Monomeric formaldehyde and hemiformals react rapidly with hydroxylamine hydrochloride, but the polymeric forms of formaldehyde must be hydrolyzed to the monomeric state before they can react. The concentration of free formaldehyde in a resin is equal to that of formaldehyde in its polymeric form. The hydrolysis of these polymers is catalyzed by hydrogen ions.

### 2.6. Measurements of Formaldehyde Emissions

To measure the formaldehyde emissions from the hardened resin, a standard test method, PV 3925 [19], was applied. The test consists of heating a determined quantity of resin (3 g), which is then placed in a filter bag and hung on a metal hook in a 500 mL polypropylene bottle filled with 50 mL of deionized water. The bottle is then placed in a ventilated oven at 60 °C for 3 h and then left at room temperature for 1 h. The emitted formaldehyde content was measured with a UV-VIS spectrophotometer at 412 nm. A calibration curve of the Hantzsch reaction product of formaldehyde, ammonia, and acetyl acetone in an acidic medium was prepared with standard formaldehyde, acetyl acetone, ammonium acetate, and acetic acid solutions. The formaldehyde emissions were calculated by fitting the absorbance on the calibration curve.

The emissions of formaldehyde were also measured in a special emission chamber using the ISO 16000-9:2006 standard [20], which specifies a general laboratory test method for determination of the area-specific emission rate of volatile organic compounds (VOCs) from products under defined climate conditions. The testing was performed after 28 days of storage under controlled conditions in the testing chamber. Measurements were carried out using stone wool impregnated with resins. The analysis was performed by a certified external laboratory.

### 2.7. Extraction of Phenolated Kraft Lignin

The samples of softwood Kraft lignin and activated softwood Kraft lignin for GPC and IR analysis were prepared according to the method described by Gan and Pan [9]. A total of 12 g of the sample was added to 30 g of H_2_O, and the pH was adjusted to 1 using concentrated HCl. Then, 30 mL of diethyl ether was added, and the mixture was centrifuged to separate the organic and aqueous phases. The insoluble fraction was further washed with ether to remove unreacted phenol and dried at 40 °C until a constant weight was achieved. In this manner, phenolated Kraft lignin was obtained and analyzed by GPC and IR as described below. The yield (%) is defined as the percentage of the dried weight of phenolated lignin over the dried weight of Kraft lignin.

### 2.8. Gel Permeation Chromatography (GPC)

The molecular weight distribution was determined by Gel Permeation Chromatography (GPC). The samples were dissolved in N, N-dimethylformamide (DMF) and filtered through a 0.45 µm pore size membrane filter. GPC was performed on a PerkinElmer Series 200 HPLC (PelkinElmer, Waltham, MA, USA) system equipped with a refractive index (RI) detector and a single Agilent PLgel column (5 µm, 300 × 7.5 mm): Mixed D and Phenomenex Guard 4 × 3 mm with pre-column. The column was heated to 60 °C, and the flow rate was set to 0.5 mL/min.

Although the absolute values of molecular weight parameters, such as the weight average molecular weight (Mw), number average molecular weight (Mn), and dispersity index (DPI), were not determined in this study, GPC analysis still provides valuable insight into the relative molecular weight distribution of the samples. While the GPC results are not calibrated to yield precise molar mass values without appropriate standards, the technique remains a powerful tool for assessing trends in molecular weight changes.

### 2.9. Fourier Transform Infrared Spectroscopy (FTIR)

To characterize the phenolation of lignin and the prepared resins, FTIR was performed on a Spectrum Two FTIR spectrometer (PerkinElmer, Waltham, MA, USA). The sample preparation procedure is described in Section 2.7. The spectra were recorded in the range of 4000–400 cm^−1^ with a resolution of 4 cm^−1^.

### 2.10. Tetradimer

The proportion of tetradimer, [bis(4-hydroxy-3,5-dimethylolphenol) methane], was determined by HPLC using an Agilent 1200 series column Eclipse XDB C18 (Agilent Tehnologies, Inc., Santa Clara, CA, USA) with an inner diameter of 4.6 mm and a length of 150 mm with a UV detector. The mobile phases used were 0.1 M HCOOH prepared with MQ water and acetonitrile (ACN). The flow rate was set to 0.75 mL/min, with the gradient flow characteristics of acetonitrile increasing from 10 to 50% and then decreasing back to 30% and 10%. Each run was set for 30 min, and the column was heated to 35 °C. A calibration curve was prepared with a pure tetradimer sample obtained from the precipitate from a resin that we produced in-house. The calibration curve was prepared for tetradimer concentrations ranging from 0 to 20%, considering that commercial resols typically contain up to 18% [1,15]. The data fit to a polynomial equation with an R2 value of 0.9981. 2,4,6-Trimethylphenol was used as an internal standard and was added to the solvent (40:60; ACN:water).

### 2.11. Mechanical Testing

For mechanical testing, sand bars were prepared by mixing 400 g of sand and 16 g of resin. The homogeneous mixtures were placed in a bar-shaped mold 120 ± 2 mm in length, 15 ± 0.5 mm in width, and 10 ± 0.5 mm in height, as per the standard ISO 3167 [21]. The mold was then placed in a preheated oven at 220 °C for 75 min. The mechanical strength of the sand sticks was measured with a Zwick Roell Z050 device (TC—FRO5OTH.A1K, serial no. 154670, ZwickRoell GmbH & Co, Germany), according to DIN 51233 [22], with a maximum force of 50 kN, which determines the maximum bending force applied to the sand bars. The mechanical strength determined in this study represents the flexural strength.

### 2.12. Activation of Kraft Lignin

To study the activation with the phenolation of Kraft lignin, several experiments were performed. The reaction was carried out under different conditions, including different temperatures, times of activation, ratios of phenol/Kraft lignin, and concentrations/compositions of the initial phenolate, as shown in Table 1.

Lignin was used to partially substitute phenol at phenol/Kraft lignin ratios of 2, 3.33, and 10, corresponding to 50%, 30%, and 10% lignin by weight relative to phenol. This substitution range was investigated to determine the optimal balance between the final resin performance and the benefits offered by lignin. In this study, lignophenols are labeled as LP X–Y–Z, where X (X = 10, 30, 50) is the percentage of lignin relative to phenol, Y indicates the solvent addition (0—no solvent; W—water as solvent; and Et—ethanol as solvent), and Z represents the lignin activation time in hours.

First, phenol was added to a glass flask and 6% inorganic catalyst was added relative to the total weight of phenol. The resulting phenolate was heated to 75 °C. Excess catalyst was added, ensuring the amount was sufficient for both the lignin activation process and subsequent synthesis of resole resin. Lignin (67% by dry weight) was added gradually to pure phenol over one hour, to ensure homogeneous dispersion and to prevent agglomeration during mixing. The reaction mixture was kept at 75 °C for one to six hours to complete the activation of lignin. The mixture was cooled to 60 °C, and the synthesis of resole resin was performed as described in Section 2.13. In cases where the effect of solvent addition was investigated, 140 g of water or ethanol (Table 1) was added to the reaction mixture. In all cases with ethanol as a solvent, an additional step of vacuum distillation was included after lignin activation to remove the remaining alcohol from the reaction mixture.

### 2.13. Synthesis of Resol Resin with Activated Lignin

Resole resin synthesis with activated softwood Kraft lignin was carried out in two consecutive steps, without interruption or the isolation of intermediate phases.

After the activation of lignin, as described in Section 2.12, the synthesis of phenol–formaldehyde resin followed. In all cases, the total amount of formaldehyde added to the flask was calculated according to a predetermined phenol-to-formaldehyde molar ratio. The amount of NaOH (50%) added was 6% relative to the total weight of phenol. In this second step, formaldehyde was added gradually over a one-hour period. The temperature of the reaction was maintained between 50 and 60 °C until the free phenol reached below 0.7% in weight, and the dilutability in the water was still unlimited (1:25). To lower the free formaldehyde concentration, in all cases, urea was added as a scavenger at a ratio of 1.2 mol of urea to 1 mol of free formaldehyde. Urea was reacted with formaldehyde for 1 h between 40 and 50 °C. When the reaction was completed, the resin was externally cooled with cold water to 20 °C. The resulting lignoresol resins were labeled as LR X–Y, where X (X = 10, 30, 50) is the percentage of lignin relative to phenol and Y indicates the solvent addition (0—no solvent; W—water as solvent; and Et—ethanol as solvent).

### 2.14. Reference Resole Resins

As a reference, two standard resole resins, prepared without lignin, were synthesized following the standard alkaline-catalyzed procedure applied in the synthesis of the resole-type phenolic resin subsequently used as a binder in mineral wool thermal insulation materials. The synthesis conditions were the same to those applied for resins with activated lignin (e.g., temperature, time, concentration of catalyst). Reference resol1 (RefR1) was prepared on a laboratory scale (1–2 kg), and reference resol2 (RefR2) was prepared on a production plant (24 tones), to compare the process parameters and final characteristics with those of the samples incorporating activated lignin. This approach allowed us to evaluate the impact of lignin activation on the overall performance and quality of the final resin.

## 3. Results and Discussion

### 3.1. Fourier Transform Infrared Spectroscopy (FTIR) of Kraft Lignin and Lignophenols

The FTIR spectra of lignins are complex because many frequencies superimpose, causing a broadening of absorption bands (below 1700 cm^−1^). Softwood Kraft lignin, used in our study, is derived from guaiacyl alcohol (G), with some contribution of sinapyl (S) and *p*-hydroxyphenyl (H) alcohol units [1,2,3], leading to additional overlap of absorption bands. As detailed by Faix [23], only a few bands can be unequivocally assigned, while the rest can be interpreted in various ways. We assigned the FTIR spectra according to known literature data, with the main purpose being to confirm lignin phenolation and resin formation, respectively, and to evaluate structural differences or similarities between standard resin and the resins with incorporated lignin.

**Figure 1:**
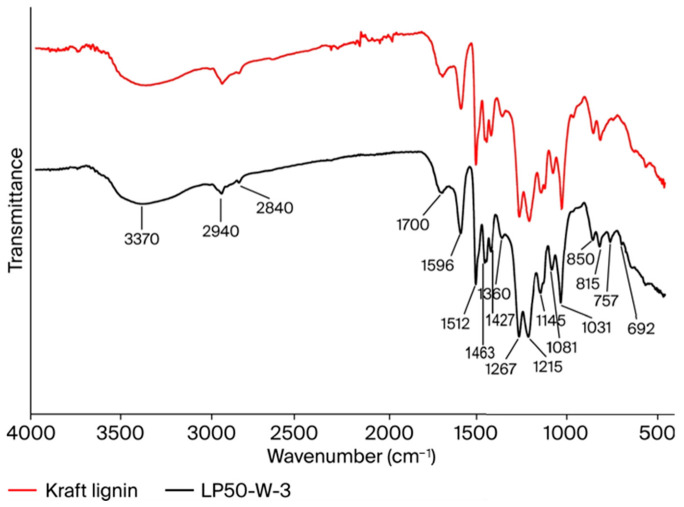
FTIR spectra of Kraft lignin and lignophenol.

The spectra of Kraft lignin and its phenolated analog are presented in Figure 1. Comparison of the two spectra shows their similarity, indicating that phenolation does not significantly affect the lignin structure. The difference between the two spectra can be observed at low wavenumbers and in the intensities of most of the bands. In LP 50-W-3, new bands at 757 and 692 cm^−1^ indicate increased infrared activity of aromatic rings due to the binding of phenol to lignin. Most of the intensities of the remaining bands are higher in LP 50-W-3 than in Kraft lignin, which is also a consequence of the binding of phenol to the lignin molecule and thus the increasing density of IR-active functional groups, or group vibrations of aromatic rings. In the following, we present a tentative assignment of the bands. The aforementioned new band at 757 cm^−1^ is a typical example of differences among the compared spectra, which is otherwise well documented in the literature. Stark et al. [24] assigned the band at 748 cm^−1^ as CCH wag; Zhao et al. [25] stated that the peak at 755 cm^−1^ belongs to linkages between phenol in the ortho or para position and α-hydroxyl groups in the side chain of lignin. Podschun et al. [26] attributed the band at 754 cm^−1^ to aromatic and C-H vibrations, while Liu et al. [27] identified the band at 757 cm^−1^ as C-H out-of-plane bending in substituted phenol. In a report on the spectroscopy of mono- and disubstituted benzene rings, Smith [28] states that the band at 690 ± 10 cm^−1^ belongs to the deformation vibration of the benzene ring, so the band at 692 cm^−1^ can be attributed to this vibration. The bands at 815 and 850 cm^−1^, according to Faix [23], are to out-of-plane C-H vibrations of the guaiacyl-type aromatic ring.

The bands above 1000 cm^−1^ are described below. In this part of the spectrum, the dominant contribution of the G unit of lignin is evident from some of the vibration bands. The following bands are assigned according to the comprehensive analysis given by Faix [23] and Zhang et al. [29]: 1031 cm^−1^ aromatic C-H in-plane deformation, G > S; plus C-O deformation in primary alcohols; plus C=O stretch; 1081 cm^−1^ C=O deformation in secondary alcohols and aliphatic ethers. The band around 1145 cm^−1^ was assigned as aromatic C-H in-plane deformation, 1215 cm^−1^ as C-C plus C-O plus C=O stretch of the guaiacyl unit, and the band at 1267 cm^−1^ as guaiacyl-type aryl ring breathing with C=O stretching [23,29].

The bands between 1360 cm^−1^ and 1512 cm^−1^ belong to a combination of different vibrations: 1360 cm^−1^ aliphatic C-H stretch in CH_3_ not in OMe, 1427 cm^−1^ aromatic skeleton vibration combined with C-H in-plane, 1463 cm^−1^ C-H deformation; asymmetric in -CH_3_ and -CH_2_- [23,29]. The band at 1512 cm^−1^ is assigned as aromatic skeletal vibrations; G > S again shows a predominant share of G units [23,24,29]. The band at 1596 cm^−1^ is ascribed to aromatic skeletal vibrations plus C=O stretch [23,24,29]. Around 1700 cm^−1^, C=O stretches of unconjugated ketone, carboxyl, and ester groups have been detected [23,24,29]. Absorption bands in the range of 2840–2940 cm^−1^ are ascribed to C-H stretching of methyl and methylene groups. Broad absorption bands around 3370 cm^−1^ are ascribed to the O-H stretching of phenolic OH and methylol groups.

### 3.2. FTIR of Resole Resin and Lignoresols

**Figure 2:**
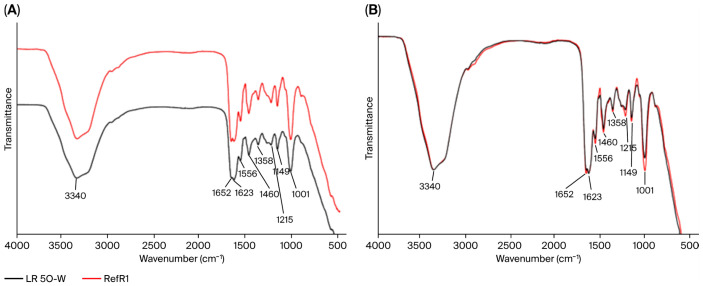
FTIR spectra of reference resol (RefR1) and lignoresol (LR 50-W) (**A**), and their normalized FTIR spectra (**B**).

The FTIR spectra of RefR1 and LR 50-W qualitatively confirm the cross-linking reactions (Figure 2). The spectra of both resins are identical in terms of frequencies, suggesting that the two resole resins have similar molecular structures. However, differences can be observed in the intensities of a number of bands, as shown in the normalized spectra of both resins (Figure 2B). The spectra are normalized to the stretching vibration of the hydroxyl groups at 3340 cm^−1^. Small peaks at 2954 and 2842 cm^−1^ are assigned to the asymmetric and symmetric CH2 stretching vibrations, respectively. The bands below 1700 cm^−1^ are tentatively assigned based on previous literature reports [6,30,31,32,33,34,35,36,37]. As mentioned above and as detailed Faix [23], the identification of the FTIR spectra of lignin and consequently of resins containing it is somewhat ambiguous due to the overlapping of absorption bands, which may be interpreted in various ways. Inspection of a number of published resole FTIR spectra reveals that the bands in the region 1652–1460 cm^−1^ [6,30,31,32,33,34,35,36,37] could be ascribed to various modes of aromatic ring vibrations, mainly with a contribution of C=C vibrations. The conditions for the synthesis of resole resins in these contributions are different, with temperatures ranging mostly between 70 and 90 °C, but also 60 °C [35] and 120 °C [33]. At lower temperatures, the reactions primarily involve the addition of formaldehyde to phenol, resulting in the formation of a series of methylol phenol monomers; at higher temperatures, however, the condensation of these monomers into dimer units is more pronounced. As a result, the number of substituents and their relative positions on the aromatic ring change [2]. Consequently, the vibration frequencies of the aromatic ring also change [23], as shown above. The band at 1358 cm^−1^ could be ascribed to phenolic OH in plane deformation [31], and 1215 cm^−1^ could be attributed to the presence of phenolic hydroxyl ether bonds [37]. Aromatic CH deformation appeared at 1149 cm^−1^ [32], and the intense band at 1001 cm^−1^ is attributed to the C-O vibration of the methylol group formed after reaction with formaldehyde. This band with the same assignment is described by other authors at 1010 cm^−1^ [30], 998–1025 cm^−1^ [32], 1024 cm^−1^ [23], and 1011.5 cm^−1^ [37].

In LR 50-W, all the absorption bands below 1700 cm^−1^ have lower intensity compared to RefR1 (Figure 2B), with the greatest difference in the C-O vibration of the methylol group at 1001 cm^−1^. The amount of formaldehyde in the synthesis of both resins was the same, but the concentration of the resulting methylol groups in LR 50-W was lower, or rather diluted, due to the presence of lignophenol, which reduces the intensity of the methylol group vibration.

**Figure 3:**
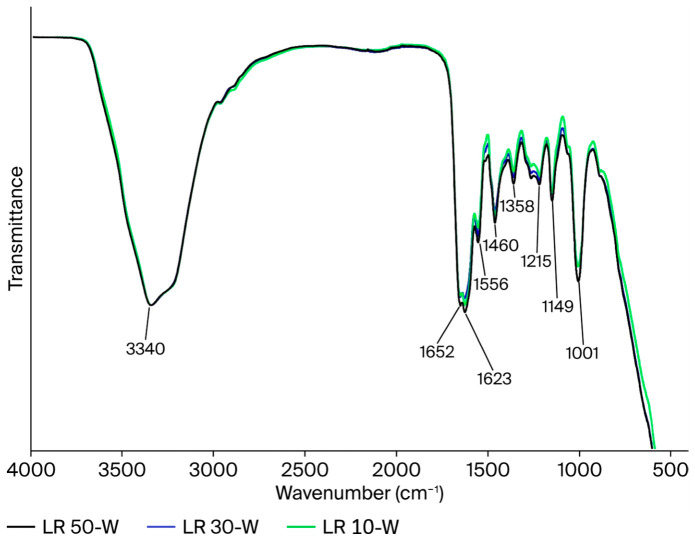
FTIR spectra of lignoresols with different lignin substitution.

The FTIR spectra of LR 10-W, LR 30-W, and LR 50-W are shown in Figure 3. The spectra of all three lignoresols are almost identical; the only differences between them are in the intensity of individual bands. This proves that, in all three cases, the same molecular structure is formed during reactions with formaldehyde regardless of the lignin–phenol ratio in lignophenol. The intensities of the FTIR bands below 1700 cm^−1^ are slightly higher in LR 50-W, indicating a larger number of functional groups available for IR absorption. This could be the consequence of changes in the functionality of phenol, formaldehyde, and lignin during the reactions.

### 3.3. Gel Permeation Chromatography (GPC)

**Figure 4:**
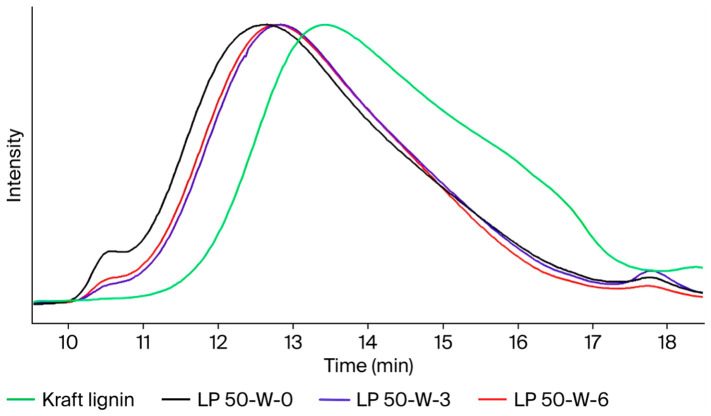
GPC chromatogram of Kraft lignin and lignophenols (LP 50-W-0, LP 50-W-3, and LP 50-W-6) at different activation times.

Figure 4 shows the GPC spectra of Kraft lignin phenolation as a function of phenolation time at a phenol/lignin ratio of 2 and their comparison with the GPC spectrum of the starting lignin. In all stages of phenolation, the main maximum present in Kraft lignin is shifted to higher molecular weights, which can be attributed to the binding of phenol to the lignin molecule. The GPC spectrum labeled as LP 50-W-0 was recorded after the gradual one-hour addition of lignin to the hot phenolate solution (Section 3.1). Of course, the reaction also proceeded during this time, which is clearly visible in the shift in the GPC curve for LP 50-W-0 to higher molecular masses (Figure 4). This GPC curve also has a smaller maximum at high molecular weights (10.6 min) and a smaller phenol peak at 17.8 min. The increase in yield to 127% further confirms the incorporation of phenol into the lignin structure (Table 2). After 3 h of activation (LP 50-W-3), a decrease in the molecular weight is observed, indicating that the activated lignin has partially depolymerized. The degradation into smaller molecules is also reflected in the decrease in the yield to 106% (Table 2).

The shifts in molecular weights indicate that the reactions of lignin activation by phenolation proceed along two opposing, competing pathways. The initial reaction of phenol binding to the lignin molecule is rapid, as the molecular weight increases immediately at the beginning of the reaction. A more detailed comparison of the GPC curves at the beginning (LP 50-W-0) and after 3 h of activation (LP 50-W-3) shows that not only is the main maximum shifted towards lower molecular weights, but the proportion of high molecular weight species also decreases (10.6 min) and the proportion of low molecular weight species increases (17.8 min). After an additional 3 h of activation (LP 50-W-6), a minimal increase in molecular weights is observed, indicating that slight repolymerization of activated lignin has occurred. At the same time, the proportion of low molecular weight species decreases slightly (17.8 min) and the proportion of high molecular weight species increases (10.6 min). Despite the continued depolymerization of some parts of the lignin, reactive fragments generated during the depolymerization process, and unreacted phenol, react with each other, leading to repolymerization and the formation of slightly larger molecules. This is also reflected in a minimal increase in yield to 108% in the case of LP 50-W-6 (Table 2). Minimal changes between LP 50-W-3 and LP 50-W-6 are also evident in Table 1, where the concentration of free phenol and viscosity changed only slightly.

As previously mentioned, the FTIR spectra of lignin and of its phenolated analogs show a high structural similarity, with some differences in the region of aromatic vibrations, where the bonding of phenol takes place. This means that, under our experimental phenolation conditions, especially low temperatures (75 °C), rearrangement reactions occur primarily in the lignin macromolecule, creating active centers for further reaction with formaldehyde.

**Figure 5:**
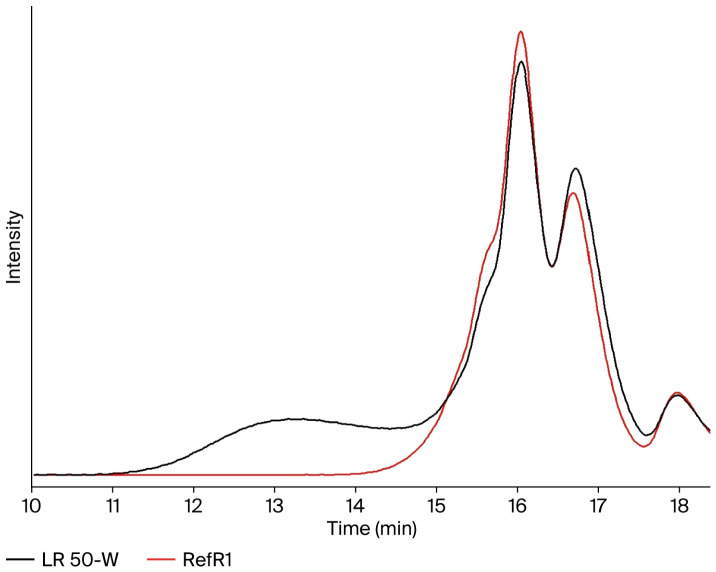
GPC chromatogram of reference resol (RefR1) and lignoresol (LR 50-W).

Figure 5 shows the GPC spectra of RefR1 and LR 50-W. The most prominent change is observed in the peak at a low retention time (11–14 min), which is present for the LR 50-W sample and absent for RefR1. This high molecular weight fraction is derived from the presence of lignin, but, as shown by the FTIR spectra (Figure 2), its molecular structure was transformed during the reaction with formaldehyde into a structure that is very similar to that of the standard resin.

### 3.4. Analysis of Process Parameters for Phenolation and Condensation

One of the main challenges was to maintain a low viscosity of the final resin to ensure good penetration properties in thermal insulation manufacturing, while increasing the lignin content in the formulation. The goal was to develop a resin with the highest possible lignin content without compromising the final resin’s properties.

The experimental parameters for the activation of lignin are summarized in Table 1. As previously mentioned in Section 3.1, the concentration of free phenol was measured after the addition of lignin at 75 °C was completed. In all samples, the concentration of free phenol increased during the phenolation reaction. The explanation for this effect can be found in the detailed description of the GPC curves for lignin activation (Figure 4). During the one-hour addition of lignin to the hot phenolate solution, the molecular weight of the lignin macromolecule increased significantly due to the binding of phenol to lignin. As the activation reaction continued, the phenolic hydroxyl groups (Ph-OH) were cleaved, which is reflected in a decrease in the molecular weight of the already-activated lignin and an increase in the concentration of free phenol. The yields of phenolized lignin at different activation times were also consistent with the course of the GPC curves (Table 2). In a recently published study [14], the authors examined the phenolation of lignin in an alkaline medium at temperatures of 95–170 °C, where they also observed an increase in the concentration of the phenolic hydroxyl (Ph-OH) groups. They also mention that no in-depth studies of alkaline phenolation have been published to date, so the reaction mechanism remains unclear.

An upward trend during the activation of lignin was also observed in viscosity measurements. In the lignophenol samples (Table 1), an increase in viscosity appeared with increasing lignin content and with increasing activation time. The dependence of viscosity on the amount of lignin is understandable because it increases the proportion of macromolecules in the reaction mixture; however, the reasons for the increase in viscosity with activation time remain unknown. When lignin is activated with concentrated phenolate (LP 50-0-3) or with phenolate diluted with ethanol (LP 50-Et-3), the viscosity increases significantly (Table 1). Although this is not favorable for the synthesis processing of resole resins, we prepared resins with all activated lignins for the purpose of comparing their properties (Table 3).

The properties of all the synthesized resole resins, including the two standard resins without lignin, are summarized in Table 3. Apart from the sample designation in the first column and the proportion of lignin in the substitution of phenol in the second column, the table shows five parameters that must be considered and fulfilled when preparing a resole resin for the production of thermal insulation products [2]. From the point of view of the production process itself, sufficiently low viscosity (ca. 50 mPa·s) is essential. The LR 50-0 viscosity of 56 mPa·s slightly exceeds the required viscosity value of 50 mPa·s, while the LR 50-Et viscosity of 100 mPa·s is significantly above the limit. In both cases, the viscosity of the intermediate activated lignin solution was already very high (Table 1), which complicated the synthesis process itself and was then also reflected in the resins’ viscosity. An additional disadvantage in the synthesis process of LR 50-Et is that ethanol needs to be removed under vacuum, which prolongs the synthesis time and increases costs. From the viscosity point of view, all resins except LR 50-0 and LR 50-Et are suitable for the manufacture of thermal insulation products. The viscosities of lignoresols with water as a solvent increase slightly with increasing lignin content. The same regularity was observed in the measurements of the viscosity of activated lignin for the same samples (Table 1 and Table 3).

The utilization of resol resin as a binder in the preparation of thermal insulation materials must have a low concentration of free phenol and free formaldehyde due to environmental emission requirements. Free phenol is typically reduced with an excess of formaldehyde, while free formaldehyde is reduced with the addition of urea. This disrupts the balance between formaldehyde and [bis(4-hydroxy-3,5-dimethylolphenol) methane] (commonly referred to as tetradimer), the latter of which can crystallize. Conventional resol resins can contain up to 18% tetradimer [15], which causes major problems in the technological process due to a reduction in resin stability and the crystallization of tetradimer. For this reason, reducing the concentration of tetradimer and preventing precipitation is a major challenge for phenolic resin manufacturers. There have been several methods for preventing tetradimer precipitation, but, to date, none have been reported for Kraft lignin.

**Figure 6:**
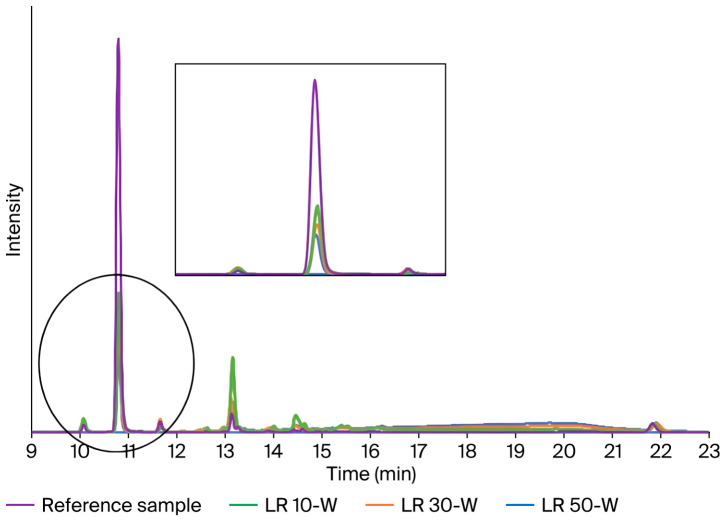
Relative concentration of tetradimer in lignoresol samples (LR 10-W, LR 30-W, and LR 50-W) with varying lignin substitutions normalized to the reference tetradimer sample (Reference sample) using HPLC analysis.

The synthesized lignin phenolic resins showed 4–11% tetradimer, while the conventional resins have 11–14%. As shown in Table 3 and Figure 6, the concentration of tetradimer decreases with an increasing initial amount of lignin. We assume that the tetradimer molecules are bonded to lignin and are not easily separated or soluble in the solvent used for analysis.

The four characteristics of the resole resins shown in Table 3, namely the concentrations of free phenol, free formaldehyde, tetradimer, and storage stability, are closely related to each other. While the concentrations of free phenol and free formaldehyde slightly decrease from LR 10-W to LR 50-W, a decrease in tetradimer content and a consequent significant increase in resin storage stability in the same direction are evident. It is obvious that, under our experimental conditions, increasing the lignin concentration has a positive effect on all the characteristics required for a resole resin used in the manufacture of thermal insulation products.

Comparison of the characteristics of all the resins with incorporated lignin with conventional resins (RefR1 and RefR2) shows the best results with LR 50-W. While the concentration of free phenol is practically the same and the concentration of free formaldehyde is slightly lower, the concentration of tetradimer in the resin is significantly lower (5–7%). As a result, the stability of this resin is greatly increased. The viscosity of this sample is also within the limits prescribed for a resol binder for thermal insulation.

### 3.5. Formaldehyde Emissions

At higher temperatures or with longer storage times, the emissions of free phenol and free formaldehyde can volatilize from the resin itself or from the thermal insulation panel. Due to the high demands of environmental restrictions and market requirements, producers of phenolic resins and their consumers—mineral wool producers—aim to minimize emissions. To determine the impact of lignin on formaldehyde emissions, analyses were conducted by two distinct approaches, allowing the results from both measurements to be directly compared and validated. Table 4, where each result represents an average of three measurements, indicates that the incorporation of lignin resulted in a reduction in formaldehyde emissions in all samples, as confirmed by both measurement techniques. The emissions from mineral wool impregnated with lignin-based resin, measured after 28 days in the environmental chamber, were 70 μg/m^3^ (LR 50-W), representing a 42% reduction compared to the mineral wool impregnated with conventional phenol–formaldehyde resin (RefR1), where the emissions were 120 μg/m^3^. When using the bottle method, the emissions for sample LR 50-W show an 83% reduction compared to the conventional phenol–formaldehyde resin. The difference between the two methods reflects the higher sensitivity of formaldehyde release detection with the bottle method, but nevertheless, with both methods, the significant potential of lignin as a phenol substitute in reducing formaldehyde emissions is visible.

The results indicate that by increasing the amount of lignin as a partial substitute for phenol in the resin formulation, the formaldehyde content decreases along with the free formaldehyde emissions from the mineral wool insulation material.

### 3.6. Mechanical Properties

Mechanical properties are important for mineral wool because they ensure stability, resistance to damage, and long-term performance in construction applications. Flexural strength provides a reliable indication of the mechanical properties of the final product produced by the mineral wool manufacturer.

**Figure 7:**
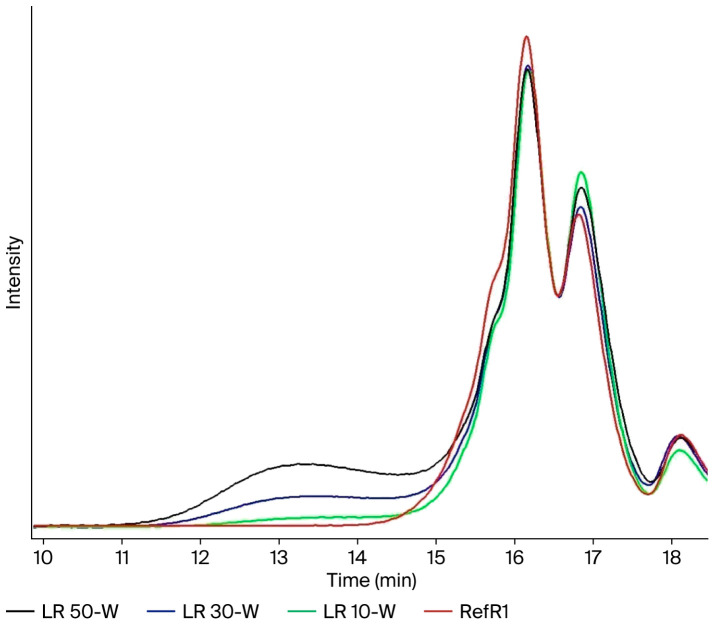
GPC chromatogram of reference resol (RefR1) and lignoresols (LR 50-W, LR 30-W, and LR 10-W) at different lignin substitutions.

The flexural strength of the synthesized resins is shown in Table 4. LR 10-W and LR 30-W have lower flexural strengths than RefR1, while LR 50-W significantly exceeds the flexural strength of the standard resin. This can be partly explained by the GPC spectra of the synthesized resole resins shown in Figure 7. All the resins have similar molecular weight distributions, which, in addition to the FTIR spectra, confirm that the cross-linking of activated Kraft lignin and formaldehyde took place (Figure 3). All the resins have their main maxima at a retention time of 16.05 min. The highest value among them is found in RefR1, with an additional shift towards higher M. This resin also has a smaller amount of lower M at 16.65 min. This distribution of molecular weights is the reason for the higher flexural strength of the standard (RefR1) resin compared to LR 10-W and LR 30-W. In the range of the highest molecular weights (11–14 min), the proportion of these M begins to increase in lignoresols, which originates from the incorporated lignin. This is clearly visible in Figure 7 and confirms the dependence of flexural strength on the amount of lignin incorporated. This proportion is large enough in LR 50-W that its flexural strength of 169 N/cm^2^ significantly exceeds the value for RefR1 of 150 N/cm^2^.

A comparison of the normalized FTIR spectra of LR 10-W, LR 30-W, and LR 50-W in Figure 3 shows differences in intensity, although the spectra are otherwise identical. All the intensities of the bands below 1700 cm^−1^ are the highest for LR 50-W. We can conclude that this resin has the highest cross-linking density, which is an additional reason for the highest flexural strength of LR 50-W.

## 4. Conclusions

The present work evaluated the activation of Kraft lignin in alkaline media at the low temperature of 75 °C, followed by a reaction with formaldehyde to produce resole resin in the same reactor without interruption or the isolation of intermediate phenolated lignin. The activated lignin and resole resins were characterized by Fourier transform infrared spectroscopy (FTIR) and gel permeation chromatography (GPC). The structure of the new resin was very similar to that of the standard resin, with a few differences in the distribution of molecular weights. The new resole resin has a low viscosity of 45 mPa·s, and more than double the stability and a significantly reduced concentration of tetradimer, which prevents the unwanted precipitation of this tetradimer. By incorporating 50% lignin by weight of phenol into the phenolic resin, a significant reduction in emissions and increased flexural strength were achieved. The new resin has greatly improved properties required for the manufacturing of thermal insulation products and shows successful scalability to industrial production [38].

## Figures and Tables

**Table 1:** Experimental parameters for lignophenol preparation by lignin phenolation.

Lignin Activation Conditions	Sample	W_phenol_/W_Kraft lignin_	Activation Temp. [°C]	Activation Time [h]	Free Phenol During Activation ^1,2^ [%]	Viscosity ^1^ [mPa·s]
Without additional solvent	LP 50-0-3	2	75	3	53/55	1043
Additional water	LP 10-W-3	10	75	3	38/43	9
	LP 30-W-3	3.33	75	3	44/47	23
	LP 50-W-3	2	75	3	53/56	65
	LP 50-W-6	2	75	6	53/57	70
Addition of EtOH	LP 50-Et-1	2	75	1	41/53	2000
	LP 50-Et-3	2	75	3	43/59	2160

^1^ Each value represents an average of three samples. ^2^ The first value of free phenol according to GC analysis presents phenol at the beginning of activation (0 min), and the second one presents free phenol at the end of the lignin activation phase.

**Table 2:** Yields of the phenolated Kraft lignins at different phenolation times.

Sample	Yield ^1^ (%)
Kraft lignin	100
LP 50-W-0	127
LP 50-W-3	106
LP 50-W-6	108

^1^ Yield = (mass of phenolated Kraft lignin)/(mass of raw Kraft lignin).

**Table 3:** Properties of final lignoresol and reference resol resins.

Sample Batch	W_phenol_/W_Kraft lignin_	Final Free Phenol ^1^ [%]	Final Free Formaldehyde ^1^ [%]	Amount of Tetradimer ^1^ [%]	Stability ^1^ [Days]	Viscosity of Final Resin ^1^ [mPa·s]
LR 50-0	2	0.35	0.13	6	12	56
LR 10-W	10	0.43	0.17	11	14	27
LR 30-W	3.3	0.41	0.15	7	14	36
LR 50-W	2	0.40	0.15	5	>25	45
LR 50-Et	2	0.20	0.15	4	14	100
RefR1	/	0.40	0.19	11	12	20
RefR2	/	0.30	0.17	14	9	23

^1^ Each value represents an average of 3 samples.

**Table 4:** Mechanical properties and formaldehyde emissions of the final lignoresol and reference resol resins.

Sample Batch	W_phenol_/W_Kraft lignin_	Flexural Strength ^1^ (N/cm^2^)	Emissions Bottle Method ^1^ (µg/g)	Emissions Chamber ^1^ (µg/m^3^)
LR 10-W	10	120	55	/
LR 30-W	3.3	143	29	100
LR 50-W	2	169	13	70
RefR1	/	150	75	120

^1^ Each value represents an average of three samples.

## Data Availability

The original contributions presented in this study are included in the article. Further inquiries can be directed to the corresponding author.
